# Comprehensive knowledge about HIV/AIDS and associated factors among adolescent girls in Rwanda: a nationwide cross-sectional study

**DOI:** 10.1186/s12879-023-08187-y

**Published:** 2023-06-07

**Authors:** Joseph Kawuki, Ghislaine Gatasi, Quraish Sserwanja, David Mukunya, Milton W. Musaba

**Affiliations:** 1 Centre for Health Behaviours Research, Jockey Club School of Public Health and Primary Care, The Chinese University of Hong Kong, SAR- China, Central Ave, Hong Kong; 2 Key Laboratory of Environmental Medicine Engineering, School of Public Health, Southeast University, 210009 Nanjing, Jiangsu Province China; 3 Programmes Department, GOAL, Khartoum, Sudan; 4 Department of Public Health, Busitema University, Mbale, Uganda; 5 Department of Obstetrics and Gynaecology, Busitema University, Mbale, Uganda

**Keywords:** Comprehensive knowledge, HIV/AIDS, Adolescent girls, Rwanda

## Abstract

**Background:**

Limited comprehensive knowledge of HIV/AIDS is highlighted as one of the major factors linked to the high prevalence of HIV among adolescents and young girls. Thus, it is crucial to identify factors that facilitate or hinder adolescent girls from having comprehensive knowledge of HIV/AIDS. We, therefore, assessed the prevalence of comprehensive knowledge about HIV/AIDS and associated factors among adolescent girls in Rwanda.

**Methods:**

We used secondary data from the Rwanda Demographic and Health Survey (RDHS) 2020 comprising 3258 adolescent girls (aged 15 to 19 years). Comprehensive knowledge was considered if an adolescent girl answered correctly all the six indicators; always using condoms during sex can reduce the risk of getting HIV, having one sexual partner only who has no other partners can reduce the risk of getting HIV, a healthy-looking person can have HIV, can get HIV from mosquito bites, can get HIV by sharing food with persons who have AIDS, and can get HIV by witchcraft or supernatural means. We, then, conducted multivariable logistic regression to explore the associated factors, using SPSS (version 25).

**Results:**

Of the 3258 adolescent girls, 1746 (53.6%, 95%CI: 52.2–55.6) had comprehensive knowledge about HIV/AIDS. Adolescent girls with secondary education (AOR = 1.40, 95% CI: 1.13–3.20), health insurance (AOR = 1.39, 95% CI: 1.12–1.73), a mobile phone (AOR = 1.26, 95% CI: 1.04–1.52), exposure to television (AOR = 1.23, 95% CI: 1.05–1.44), and a history of an HIV test (AOR = 1.26, 95% CI: 1.07–1.49) had higher odds of comprehensive HIV knowledge, compared to their respective counterparts. However, girls residing in Kigali (AOR = 0.65, 95% CI: 0.49–0.87) and Northern (AOR = 0.75, 95% CI: 0.59–0.95) regions, and those of Anglican religion (AOR = 0.82, 95% CI: 0.68–0.99) had less odds of comprehensive knowledge compared to those in Southern region and of the Catholic religion.

**Conclusions:**

To increase the comprehensive understanding of the disease at a young age, the need for expanded access to HIV preventive education through formal educational curriculum, and mass and social media via mobile phones is highlighted. In addition, the continued involvement of key decision-makers and community actors, such as religious leaders, is vital.

## Introduction

Globally, Human Immunodeficiency Virus and Acquired Immune Deficiency Syndrome (HIV/AIDS) remains a public health concern and sub-Saharan Africa (SSA) has the greatest burden [1–3]. Over 38 million people are infected with HIV worldwide, with women making up more than half (19.2 million) of the infected. Sub-Saharan African women account for 15.9 million of the 19.2 million total [4, 5]. Young women and teenage girls account for 25% of new infections in SSA [4, 5]. Eastern and Southern African women face the highest burden of HIV contributing to three in five new infections, while young women aged 15 to 24 years are almost three times more likely than their male peers to acquire HIV infection [4, 5]. It is estimated that over 700 adolescents acquire HIV daily and over 360,000 are expected to die of HIV/AIDS-related illnesses between 2018 and 2030 [6]. Adolescent HIV/AIDS-related mortality in Africa remains significant and AIDS is among the leading causes of death among African adolescents with SSA having the highest AIDS-related mortality burden [7–9].

Rwanda’s adult (15 to 64 years) HIV prevalence is 3% with women facing a higher burden compared to men [5, 10]. Although efforts such as screening blood donations, increased support for diagnostic services, health education, prevention services such as free condoms and scaling-up of antiretroviral therapy (ART) have greatly reduced HIV incidence in Rwanda, there exist strong gender disparities in terms of HIV prevalence and incidence [11–13]. The Rwanda Demographic and Health Survey (RDHS) 2020 further reports that 9% of Rwandan women engage in sexual intercourse with someone else other than their partner. Of these women having multiple sexual partners, only 45% reported having used a condom during their last sexual intercourse [14]. Societal attitudes and misperceptions about sexuality education have made access to adolescent-friendly, timely and appropriate health education, including comprehensive HIV knowledge a common challenge in many SSA countries including Rwanda [7, 15, 16]. This further exposes adolescents to other unreliable sources of information [15].

Limited comprehensive knowledge of HIV has been documented as one of the major factors linked to the high prevalence of HIV among adolescents and young women (AGYW) [2, 17]. Furthermore, limited HIV knowledge among HIV-positive adolescents also risks a lack of ART adherence, while among the HIV-negative ones, it may lead to increased stigma towards HIV-infected peers, which stigma may hinder many from testing and adhering to ART [7, 18]. Having adequate comprehensive HIV knowledge has been shown to enhance adolescents’ and young women’s ability to negotiate for safer sex hence helping to reduce the risk of contracting HIV [2, 19].

Although efforts aimed at improving comprehensive HIV knowledge among the young and sexually active population have been prioritized, not much success has been registered [2, 7]. Factors such as early marriages, and patriarchal practices that favour boys over girls in terms of accessing education have predisposed teenage girls and young women in SSA to a higher risk of contracting HIV than their male peers [2, 20]. This is made worse by the fact that sexual and reproductive health (SRH) services in Rwanda seem to have insufficient involvement of adolescents in service provision [21, 22]. Moreover, regional studies have also shown healthcare providers’ HIV knowledge scores to be low, with Rwanda scoring lower than the other countries [23].

Nonetheless, there is a dearth of information regarding comprehensive knowledge about HIV/AIDS among adolescents with most of the available HIV/AIDS studies focusing on prevalence, incidence and ART uptake/adherence [3, 8, 10, 11, 13]. In Rwanda, only two studies have recently assessed HIV knowledge and these focussed on healthcare workers and men [12, 23], with none done among adolescents. This creates uncertainty about whether the previous studies’ findings equally apply to women and adolescent girls in Rwanda who tend to have different risks and dynamics.

We, therefore, examined the prevalence of comprehensive knowledge about HIV/AIDS and associated factors among adolescent girls in Rwanda, using a nationally representative dataset. It is crucial to understand and address HIV knowledge gaps among adolescents, since ensuring comprehensive knowledge of HIV/AIDS at an early age appears to be more beneficial as opposed to later in life when the effects of poor/low knowledge of the disease may be irreversible. Moreover, the results of this study would be vital for designing better and more evidence-based behavior change interventions.

## Methods

### Study sampling and participants

The dataset used was from the 2019-20 Rwanda Demographic Survey (RDHS), which was a cross-sectional study and employed a two-stage sample design. The first stage involved cluster selection consisting of enumeration areas (EAs), while the second stage involved systematic sampling of households in all the selected EAs leading to a total of 13,005 households [14]. In particular, the data used in this analysis were from the household and the woman’s questionnaires.

During this survey (RDHS), the data collection period was from November 2019 to July 2020, which took longer than expected due to the COVID-19 pandemic restrictions [1]. Women aged 15–49 years who were either permanent residents of the selected households or visitors who stayed in the household the night before the survey were eligible to be interviewed. Of the 13,005 households that were selected for the survey, 12,951 were occupied and 12,949 were successfully interviewed giving a response rate of 99.9% [14]. The eligible sample was 14,675 women aged 15–49, but 14,634 women were successfully interviewed leading to a 99.7% response rate [14]. For this analysis, we considered only adolescent girls aged 15–19 years interviewed during the survey, and of the selected households, whose sample was 3,258.

### Variables

#### Dependent variables

The study outcome variable was comprehensive knowledge about HIV/AIDS, which was a composite variable scored from six yes/no questions; (1) Always using condoms during sex can reduce the risk of getting HIV, (2) Having only one sexual partner, who has no other partners can reduce risk of getting HIV, (3) A healthy looking person can have HIV, (4) Can get HIV from mosquito bites, (5) Can get HIV by sharing food with a person who has AIDS, and (6) Can get HIV by witchcraft or supernatural means [24, 25]. Comprehensive knowledge was considered if an adolescent girl answered all six questions correctly; that is, (“Yes” for questions 1,2, and 3, and “No” for questions 4,5, and 6).

#### Explanatory variables

Based on the available literature and data, we included possible determinants of comprehensive knowledge about HIV/AIDS [24–28]. Eighteen (18) variables were considered and of these, two were community-level factors that included; place of residence (categorized as rural and urban), and region of residence (Kigali, South, West, East and North). Four household-level factors included; household size (classified into “less than six” and “six and above”), sex of household head (female and male), wealth index (categorized into five quintiles that ranged from the poorest to the richest quintile), and health insurance (yes and no). Wealth index was calculated by RDHS from information on household asset ownership using Principal Component Analysis [14]. Twelve (12) individual-level factors were also considered in the analysis, including; age (categorized as 15, 16, 17, 18 and 19 years), educational level (no education, primary, secondary and tertiary), working status (working and not working), marital status (married and unmarried), religion (Catholic, Protestant, and others), history of having an STI in last 12 months (yes and no), and exposure to news, radio and television (yes and no), contraceptive use (yes and no), having done an HIV test before (yes and no) and owning a mobile phone (yes and no).

### Statistical analysis

In this analysis, we applied the DHS sample weights to account for the unequal probability sampling in different strata and ensure the representativeness of the study results [29, 30]. We used Statistical Package for Social Science’s (SPSS) (version 25.0) complex samples package incorporating the following variables in the analysis plan to account for the multistage sample design inherent in the RDHS dataset: individual sample weight, sample strata for sampling errors/design, and cluster number [24, 29]. Initially, we did descriptive statistics for both dependent and independent variables, where frequencies and proportions/percentages for categorical dependent and independent variables have been presented. We, then, conducted bivariable logistic regression to assess the association of each independent variable (i.e. selected socio-demographic factors) with comprehensive HIV knowledge and crude odds ratio (COR), 95% confidence interval (CI) and p-values are presented. Independent variables found significant at the bivariable level with p-values less than 0.25 were then included in the multivariable model. Moreover, independent variables that were non-significant at the bivariable analysis level but were associated with comprehensive knowledge in previous studies were also included in the multivariable logistic regression model. The final model controlled for all the included variables/factors where we calculated and presented their respective adjusted odds ratios (AOR), 95% confidence intervals (CI) and p-values, at a statistical significance level of 0.05. All selected variables in the model were assessed for multi-collinearity, which was considered present if the variables had a variance inflation factor (VIF) greater than 10 [31]. However, none of the variables had a VIF above 3.

## Results

We used a weighted sample of 3258 adolescent girls in this study, of which about half had primary education (50.6%), the majority were not working (61.8%), unmarried (97.7%), had health insurance coverage (82.1%), and owned a mobile phone (74.1%). Moreover, a majority had no history of STI (99.6%), were not using contraceptives (97.1%), and had never done an HIV test before (63.5%). Regarding media use, the majority were not exposed to newspapers (62.9%) and television (50.2%) but had radio exposure (82.7%). In addition, the majority of the respondents were from male-headed households (67.8%) of 6 and more members (55.3%), and resided in rural areas (82.2%), as detailed in Table 1.

Regarding knowledge about HIV/AIDS, the majority individually answered correctly all six knowledge questions (range 77.8–94.9%). However, the prevalence of comprehensive knowledge (those who answered all six questions correctly) was 53.6% with a 95% confidence interval of (52.2-55.6%), as shown in Table 2.

**Table 1:** Background characteristics of Rwandan adolescents aged 15 to 19 years as per the 2020 RDHS

Characteristics	Frequency (%), N = 3258
**Age (years)**	
15	810(24.9)
16	680(20.9)
17	667(20.5)
18	504(15.5)
19	596(18.3)
**Educational level**	
Tertiary	13(0.4)
Secondary	1564(48.0)
Primary	1650(50.6)
No education	32(1.0)
**Working status**	
Working	1246(38.2)
Not working	2012(61.8)
**Marital status**	
Married	73(2.3)
Unmarried	3185(97.7)
**Religion**	
Catholic	1242(38.1)
Protestant	1517(46.6)
Others	500(15.3)
**Health insurance coverage**	
Yes	2676(82.1)
No	582(17.9)
**History of STI**	
Yes	14(0.4)
No	3244(99.6)
**Exposure to newspapers**	
Yes	1208(37.1)
No	2050(62.9)
**Exposure to radio**	
Yes	2694(82.7)
No	564(17.3)
**Exposure to television**	
Yes	1624(49.8)
No	1635(50.2)
**Contraceptive use**	
Yes	94(2.9)
No	3165(97.1)
**HIV test**	
Yes	1189(36.5)
No	2070(63.5)
**Owns mobile phone**	
Yes	845(25.9)
No	2414(74.1)
**Wealth index**	
Richest	814(25.0)
Richer	678(20.8)
Middle	650(19.9)
Poorer	619(19.0)
Poorest	497(15.2)
**Household size**	
Less than 6	1458(44.7)
6 and above	1801(55.3)
**Sex of household head**	
Male	2210(67.8)
Female	1049(32.2)
**Residence**	
Urban	579(17.8)
Rural	2680(82.2)
**Region**	
Kigali	397(12.2)
West	694(21.3)
East	989(30.3)
North	497(15.2)
South	681(20.9)

**Table 2:** Knowledge about HIV/AIDS, as per the 2020 Rwanda Demographic Health Survey, *N* = 3258

Knowledge item	Yes (%)	No (%)
Always using condoms during sex can reduce the risk of getting HIV ^a^	2902(89.1)	324(9.9)
Having 1 sex partner only, who has no other partners can reduce the risk of getting HIV ^a^	2799(85.9)	427(13.1)
A healthy-looking person can have HIV ^a^	2537(77.8)	689(21.1)
Can get HIV from mosquito bites ^a^	352(10.8)	2874(88.2)
Can get HIV by sharing food with a person who has AIDS ^a^	289(8.9)	2937(90.1)
Can get HIV by witchcraft or supernatural means ^a^	135(4.1)	3091(94.9)
**Comprehensive knowledge of HIV/AIDS** ^a^	**1746 (53.6%), (95%CI: 52.2–55.6)**	1480(45.4)

a = missing 33 responses, HIV = Human immunodeficiency virus, AIDS = Acquired immune deficiency syndrome

### Factors associated with comprehensive knowledge of HIV/AIDS

Results of bivariable analyses are detailed in Table 3, with significant factors independently associated with comprehensive knowledge highlighted. In the adjusted logistic regression model, the factors with significant association include; educational level, religion, health insurance, exposure to television, having had an HIV test before, owning a mobile phone, and region (Table 3).

Adolescent girls with secondary education (AOR = 1.40, 95% CI: 1.13–3.20) had more odds of having comprehensive knowledge about HIV/AIDS compared to those with no education. Those with health insurance (AOR = 1.39, 95% CI: 1.12–1.73) had more odds of having comprehensive knowledge compared to their fellows with no health insurance, similar to girls with exposure to television (AOR = 1.23, 95% CI: 1.05–1.44) who also had more odds compared to those with no exposure. In addition, adolescent girls who had ever tested for HIV (AOR = 1.26, 95% CI: 1.07–1.49) had more odds of having comprehensive knowledge about HIV/AIDS compared to those who had never tested, and the same applied to those owning a mobile phone (AOR = 1.26, 95% CI: 1.04–1.52) who also had more odds compared to their counterparts with no mobile phones. In contrast, respondents from Kigali (AOR = 0.65, 95% CI: 0.49–0.87) and Northern (AOR = 0.75, 95% CI: 0.59–0.95) region had less odds of having comprehensive knowledge about HIV/AIDS compared to their fellows in the Southern region. The same applied to Protestant girls (AOR = 0.82, 95% CI: 0.68–0.99) who also had less odds of having comprehensive knowledge compared to the Catholic respondents.

**Table 3:** Factors associated with comprehensive knowledge about HIV/AIDS among adolescent girls, as per 2020 RDHS

Variable	Crude odds ratio, COR (95% CI)	p-value*	Adjusted odds ratio, AOR (95% CI)	p-value**
**Age (years)**		**< 0.01**		0.34
15	1		1	
16	1.09(0.88–1.34)		1.06(0.85–1.31)	
17	1.34(1.09–1.64)		1.23(0.99–1.53)	
18	1.38(1.10–1.72)		1.20(0.95–1.53)	
19	1.44(1.15–1.80)		1.20(0.91–1.58)	
**Educational level**		**< 0.01**		**< 0.01**
No education	1		1	
Primary	0.93(0.42–2.08)		0.82(0.36–1.89)	
Secondary	1.77(1.48–3.92)		**1.40(1.13–3.20)**	
Tertiary	7.79(0.81–74.93)		5.21(0.54–50.43)	
**Working status**		**0.01**		0.30
Not working	1		1	
Working	0.81(0.69–0.94)		0.91(0.76–1.09)	
**Marital status**		0.59		0.65
Unmarried	1		1	
Married	1.15(0.68–1.95)		1.14(0.64–2.04)	
**Religion**		**0.04**		**0.03**
Catholic	1		1	
Protestant	0.82(0.69–0.98)		**0.82(0.68–0.99)**	
Others	1.06(0.84–1.33)		1.00(0.80–1.26)	
**Health insurance coverage**		**< 0.01**		**< 0.01**
No	1		1	
Yes	1.61(1.32–1.97)		**1.39(1.12–1.73)**	
**History of STI**				
No	1	**0.06**	1	0.06
Yes	4.39(0.92–20.95)		4.45(0.94–21.07)	
**Exposure to newspapers**		**0.02**		0.11
No	1		1	
Yes	1.21(1.04–1.40)		0.87(0.74–1.03)	
**Exposure to radio**		**< 0.01**		0.24
No	1		1	
Yes	1.55(1.27–1.89)		1.15(0.91–1.45)	
**Exposure to Television**		**< 0.01**		**0.01**
No	1		1	
Yes	1.42(1.22–1.65)		**1.23(1.05–1.44)**	
**Contraceptive use**		0.60		0.71
No	1		1	
Yes	1.12(0.73–1.72)		0.91(0.56–1.47)	
**HIV test**		**< 0.01**		**0.01**
No	1		1	
Yes	1.44(1.23–1.69)		**1.26(1.07–1.49)**	
**Owns mobile phone**		**< 0.01**		**0.02**
No	1		1	
Yes	1.382(1.164–1.641)		**1.26(1.04–1.52)**	
**Wealth index**		**< 0.01**		0.12
Poorest	1		1	
Poorer	1.06(0.84–1.35)		0.88(0.68–1.13)	
Middle	1.34(1.03–1.75)		1.03(0.77–1.36)	
Richer	1.79(1.40–2.30)		1.18(0.90–1.56)	
Richest	1.44(1.11–1.88)		0.90(0.65–1.24)	
**Household size**		0.88		0.80
6 and above	1		1	
Less than 6	1.01(0.86–1.19)		1.02(0.86–1.22)	
**Sex of household head**		0.29		0.53
Male	1		1	
Female	0.92(0.78–1.08)		0.95(0.80–1.12)	
**Residence**		0.69		0.84
Rural	1		1	
Urban	1.05(0.84–1.30)		0.97(0.73–1.29)	
**Region**		**< 0.01**		**< 0.01**
South	1		1	
North	0.72(0.57–0.90)		**0.75(0.59–0.95)**	
East	1.07(0.87–1.32)		1.08(0.87–1.34)	
West	0.78(0.63–0.97)		0.81(0.65–1.01)	
Kigali	0.80(0.60–1.05)		**0.65(0.49–0.87)**	

**Bold** = significant, *= significant at 0.25, **= significant at 0.05, CI = confidence interval, RDHS = Rwanda demographic health survey, HIV/AIDS = Human immunodeficiency virus/Acquired immune deficiency syndrome

## Discussion

This study assessed the comprehensive knowledge among Rwandan adolescent girls. We discovered that, despite the progress made, knowledge remains low but still higher than in many other countries, with nearly half of the respondents (53.6%) correctly answering the six HIV/AIDS-related questions in the RDHS. This is consistent with a study assessing knowledge in various SSA countries, which found that Rwandan women had a greater sense of comprehensive knowledge than women in other countries in the region [32, 33]. The study’s results also indicate that Rwandan adolescent girls are relatively knowledgeable about HIV/AIDS transmission modes. Over 80% of the adolescents were well aware that using condoms consistently and having only one sex partner who has no other partners can minimize the risk of contracting HIV, which may help to reduce the number of young people participating in unsafe sexual behavior. They also understood that HIV cannot be transmitted through mosquito bites or sharing food with infected persons, demonstrating the Rwandan population’s progress in dispelling myths and misconceptions that perpetuate stigma against HIV-positive people. Nonetheless, there is room for improvement to attain higher levels of comprehensive knowledge of the disease among young people. Despite the documented link between HIV knowledge and HIV risk, adolescents and young women face other external issues that also predispose them to a higher risk of HIV, such as intimate and gender-based violence, limited access to sexual and reproductive healthcare, amongst others [2, 20, 21, 34]. HIV/AIDS education and awareness programs should thus consider and incorporate such issues for successful overall HIV prevention and reduction.

The educational level of adolescent girls, religion, health insurance, exposure to media, having had an HIV test before, owning a cellphone, and region were all found to be significantly associated with comprehensive HIV/AIDS knowledge. Education was found to have a positive influence on comprehensive knowledge of HIV/AIDS, with those having secondary-level education being more knowledgeable than those with no education. This is not surprising given similar prior findings in other countries such as Malawi [32], Kenya [35], Uganda [36], Ethiopia [37] and Ghana [38]. This stresses the importance of continuing to advocate for and invest in young girls’ and women’s literacy, which has been connected to sustainable women’s empowerment that is passed down through generations since literate women are more likely to send their children to school [39, 40].

Girls who had been exposed to television were more likely to have comprehensive knowledge than those who had not been exposed, a finding also demonstrated in earlier studies [36, 41]. One possible reason is that media exposure enhances the likelihood of coming across HIV/AIDS-related knowledge. Individuals can learn about health and health-related topics from television in a variety of forms, including music, news stories, dramas, movies, and ads, which enhances the odds of reaching people with a variety of interests. Campaigns utilizing television have been reported to be successful in developing countries [42]. As a result, an enhanced partnership between media companies and HIV/AIDS stakeholders is needed to amplify messages and tailor them toward young women and the overall Rwandan community.

Similarly, our study revealed that mobile phone ownership was linked to having a more thorough HIV/AIDS understanding than the lack of it. Mobile phones have recently become a significant part of people’s daily lives, and they are especially appealing to the youth [43, 44]. It is said to have been gradually overtaking ‘traditional media’ such as television [45]. Studies have previously demonstrated that social media can be a useful tool for health promotion [46, 47]. Thus, in addition to increasing mass media outreach, efforts should also be made to work with telecommunication companies to convey messages and to take advantage of social media platforms to spread HIV/AIDS and other health-related information, possibly in collaboration with public figures and individuals whom young women look up to as role models.

Our findings corroborate those of Ethiopian [48] and Ugandan [36] researchers who reported that adolescent girls who had ever been tested for HIV were also more likely to have comprehensive knowledge of HIV/AIDS than those who had never been tested. Counseling before and after an HIV test is essential because it provides for education, prevention of future transmission in the event of a positive result, and an understanding of the need for prompt medical attention [49]. Therefore, having had a test done indicates a person’s potential exposure to information and explains the higher HIV/AIDS comprehensive knowledge. This evidence could be used to influence decisions about capacity building and keeping counselors and healthcare providers in all HIV testing facilities up to date so that they may in turn convey accurate information.

When compared to their counterparts in the Southern region, adolescent girls living in Kigali were shown to be less likely to have comprehensive knowledge of HIV/AIDS. This is in contrast with prior studies in Burkina Faso [50], Nigeria [51], and even outside of the Sub-Saharan African context [52, 53] that reported rural residents to have less HIV-related knowledge compared to urban residents. More research needs to be conducted to fully grasp this difference in dynamics in the Rwandan context.

In many cultures, religion significantly influences how people interact and make decisions regarding their health [54]. While some religious communities have been actively involved in spreading awareness and encouraging testing [55–57], others believe that immoral sexual behavior is to blame for HIV/AIDS, which heightens the stigma attached to the disease [57, 58]. Our research revealed that Catholic respondents were more likely than protestant respondents to have comprehensive knowledge of HIV, indicating some variation in how different Christian faith groups have responded to the disease. Comprehensive awareness of HIV/AIDS within the Rwandan community would greatly benefit from the increased involvement of religious leaders of all denominations as well as teamwork and partnerships.

Adolescent girls with health insurance were also more likely to have comprehensive knowledge compared to those with no health insurance. This could be due to the fact that having access to health insurance increases the likelihood of seeking preventive care and therefore multiplying the chances to receive health information [59].

### Study strengths and limitations

Since the RDHS data used in this study is nationally representative, the results can be generalized to other Rwandan female populations between the ages of 15 and 19 years. Moreover, DHS data is also known for generating substantial data, with high response rates, high-quality interviewer training, standardized data collection processes across countries, and consistent content across time, allowing these findings to be compared across populations and over time to assess trends. However, there are some notable limitations in this study. First, since the Demographic and Health Survey is collected simultaneously, it is challenging to establish a causal link between the dependent and independent variables. As a result, our findings are bound to associations. Second, given that the DHS is self-reported, there is a chance that under-reporting invalidates our findings, especially given how sensitive the issue of HIV is to respondents. Moreover, we focussed only on Rwandan adolescents, thus future studies should consider doing a multi-country analysis to validate our findings and have more aggregated and broader insight on the topic. Despite these limitations, our findings contribute significantly to the body of knowledge and represent a valuable resource for policymakers who are implementing HIV prevention intervention programs.

## Conclusions

Adopting practices that lower the risk of HIV transmission requires a thorough understanding of HIV/AIDS. About half of the Rwandan adolescent girls had comprehensive knowledge about HIV/AIDS and this was associated with various sociodemographic factors; education level, health insurance, exposure to television, owning a mobile phone, history of HIV test, region and religion. To increase the comprehensive understanding of the disease starting at a young age, access to education on HIV prevention must be expanded through interventions including mainstreaming HIV/AIDS into the formal educational curriculum as well as additional sources like mass and social media via mobile phones. The results also showed the necessity for concerned decision-makers to continue involving other key community actors, such as religious leaders, and to expand access to health insurance.

## Data Availability

The data set used is openly available upon permission from the MEASURE DHS website (URL: https://www.dhsprogram.com/data/available-datasets.cfm). However, authors are not authorized to share this data set with the public but anyone interested in the data set can seek it with written permission from the MEASURE DHS website (URL: https://www.dhsprogram.com/data/available-datasets.cfm).

## Abbreviations

EA: Enumeration area
AOR: Adjusted Odds Ratio
HIV: Human Immunodeficiency Virus
AIDS: Acquired Immune Deficiency Syndrome
SSA: Sub-Saharan Africa
ART: Antiretroviral therapy
SRH: Sexual and reproductive health
CI: Confidence Interval
COR: Crude Odds Ratio
DHS: Demographic Health Survey
RDHS: Rwanda Demographic Health Survey
VIF: Variance inflation factor
SPSS: Statistical Package for Social Science

## References

[1] World Health Organization. Fact Sheet on HIV and AIDS. 2022. https://www.who.int/en/news-room/fact-sheets/detail/hiv-aids. Accessed 12th Aug 2022.

[2] Frimpong JB, Budu E, Adu C, Mohammed A, Tetteh JK, Seidu A-A, Ahinkorah BO. Comprehensive HIV/AIDS knowledge and safer sex negotiation among adolescent girls and young women in sub-Saharan Africa.J Biosoc Sci2021:1–13. 10.1017/S0021932021000493 34558397

[3] Kawuki J, Kamara K, Sserwanja Q (2022). Prevalence of risk factors for human immunodeficiency virus among women of reproductive age in Sierra Leone: a 2019 nationwide survey. BMC Infect Dis.

[4] Joint United Nations Programme on HIV/AIDS (UNAIDS). UNAIDS 2020 | Reference: UNAIDS Data 2020- State of the Epidemic. Geneva: UNAIDS. ; 2020. Available from: https://www.unaids.org/sites/default/files/media_asset/2020_aids-data-book_en.pdf. Accessed 12th Aug 2022.

[5] Niragire F, Ndikumana C, Nyirahabimana MG, Uwizeye D (2021). Prevalence and factors associated with fertility desire among HIV-positive women in Rwanda in the context of improved life expectancy. Archives of Public Health.

[6] UNICEF. Around 80 adolescents will die of AIDS every day by 2030, at current trends – UNICEF. https://www.unicef.org/press-releases/around-80-adolescents-will-die-aids-every-day-2030-current-trends-unicef. Accessed 12th Aug 2022.

[7] Badru T, Mwaisaka J, Khamofu H, Agbakwuru C, Adedokun O, Pandey SR, Essiet P, James E, Chen-Carrington A, Mastro TD (2020). HIV comprehensive knowledge and prevalence among young adolescents in Nigeria: evidence from Akwa Ibom AIDS indicator survey, 2017. BMC Public Health.

[8] UNICEF, Adolescent. deaths from AIDS tripled since 2000. Press release. 2015. https://www.efe.com/efe/english/life/unicef-says-adolescent-deaths-from-aids-tripled-since-2000/50000263-2775480. Accessed 12th Aug 2022.

[9] World Health Organization. Health for the world’s adolescents: a second chance in the second decade: summary. World Health Organization. ; 2014. https://apps.who.int/iris/handle/10665/112750. Accessed 12th Aug 2022.

[10] Nsanzimana S, Rwibasira GN, Malamba SS, Musengimana G, Kayirangwa E, Jonnalagadda S, Fazito Rezende E, Eaton JW, Mugisha V, Remera E (2022). HIV incidence and prevalence among adults aged 15–64 years in Rwanda: results from the Rwanda Population-based HIV Impact Assessment (RPHIA) and District-level modeling, 2019. Int J Infect Dis.

[11] Nsanzimana S, Remera E, Kanters S, Mulindabigwi A, Suthar AB, Uwizihiwe JP, Mwumvaneza M, Mills EJ, Bucher HC (2017). Household survey of HIV incidence in Rwanda: a national observational cohort study. The Lancet HIV.

[12] Rugigana E, Birungi F, Nzayirambaho M (2015). HIV knowledge and risky sexual behavior among men in Rwanda. Pan Afr Med J.

[13] Rwibasira GN, Malamba SS, Musengimana G, Nkunda RCM, Omolo J, Remera E, Masengesho V, Mbonitegeka V, Dzinamarira T, Kayirangwa E (2021). Recent infections among individuals with a new HIV diagnosis in Rwanda, 2018–2020. PLoS ONE.

[14] National Institute of Statistics of Rwanda - NISR, Ministry of Health - MOH, ICF (2021). Rwanda demographic and health survey 2019-20. In. Kigali, Rwanda and Rockville.

[15] Widman L, Choukas-Bradley S, Noar SM, Nesi J, Garrett K (2016). Parent-adolescent sexual communication and adolescent safer sex behavior: a meta-analysis. JAMA Pediatr.

[16] Sserwanja Q, Musaba MW, Mukunya D (2021). Prevalence and factors associated with modern contraceptives utilization among female adolescents in Uganda. BMC Womens Health.

[17] Siziya S, Muula AS, Rudatsikira E (2008). HIV and AIDS-related knowledge among women in Iraq. BMC Res Notes.

[18] Richard A-K, Roland YK, Christian YK, Cécile K-KA, Michel AJ, Lacina C, Vincent AK. Knowledge, Attitudes, and Practices of HIV-Positive Adolescents Related to HIV/AIDS Prevention in Abidjan (Côte d’Ivoire). International journal of pediatrics 2020, 2020:8176501–8176501. 10.1155/2020/8176501 PMC7785380 33456475

[19] De Coninck Z, Feyissa IA, Ekström AM, Marrone G (2014). Improved HIV awareness and perceived empowerment to Negotiate Safe Sex among Married Women in Ethiopia between 2005 and 2011. PLoS ONE.

[20] Mavhu W, Rowley E, Thior I, Kruse-Levy N, Mugurungi O, Ncube G, Leclerc-Madlala S (2018). Sexual behavior experiences and characteristics of male-female partnerships among HIV positive adolescent girls and young women: qualitative findings from Zimbabwe. PLoS ONE.

[21] Ndayishimiye P, Uwase R, Kubwimana I, Niyonzima JdlC, Dzekem Dine R, Nyandwi JB, Ntokamunda Kadima J (2020). Availability, accessibility, and quality of adolescent sexual and Reproductive Health (SRH) services in urban health facilities of Rwanda: a survey among social and healthcare providers. BMC Health Serv Res.

[22] Schwandt HM, Feinberg S, Akotiah A, Douville TY, Gardner EV, Imbabazi C, McQuin E, Mohamed M, Rugoyera A, Musemakweli D (2018). Family planning in Rwanda is not seen as population control, but rather as a way to empower the people”: examining Rwanda’s success in family planning from the perspective of public and private stakeholders. Contracept Reproductive Med.

[23] Pineda-Antunez C, Contreras-Loya D, Rodriguez-Atristain A, Opuni M, Bautista-Arredondo S (2021). Characterizing health care provider knowledge: evidence from HIV services in Kenya, Rwanda, South Africa, and Zambia. PLoS ONE.

[24] Teshale AB, Yeshaw Y, Alem AZ, Ayalew HG, Liyew AM, Tessema ZT, Tesema GA, Worku MG, Alamneh TS. Comprehensive knowledge about HIV/AIDS and associated factors among women of reproductive age in sub-saharan Africa: a multilevel analysis using the most recent demographic and health survey of each country. BMC Infect Dis. 2022 Dec;22(1):1–0. 10.1186/s12879-022-07124-9 PMC8822699 35130865

[25] Dadi TK, Feyasa MB, Gebre MN. HIV knowledge and associated factors among young Ethiopians: application of multilevel order logistic regression using the 2016 EDHS. BMC Infect Dis. 2020 Dec;20(1):1–1. 10.1186/s12879-020-05436-2 PMC7525965 32993536

[26] Yaya S, Bishwajit G, Danhoundo G, Shah V, Ekholuenetale M. Trends and determinants of HIV/AIDS knowledge among women in Bangladesh. BMC Public Health. 2016 Dec;16(1):1–9. 10.1186/s12889-016-3512-0 PMC4989494 27535231

[27] Efendi F, Pratama ER, Hadisuyatmana S, Indarwati R, Lindayani L, Bushy A. HIV-related knowledge level among indonesian women between 15 years and 49 years of age. Afr Health Sci. 2020 Apr;20(1):83–90. 10.4314/ahs.v20i1.13 PMC7750044 33402896

[28] Hong SY, Thompson D, Wanke C, Omosa G, Jordan MR, Tang AM, Patta S, Mwero B, Mjomba I, Mwamburi M. Knowledge of HIV transmission and associated factors among HIV-positive and HIV-negative patients in rural Kenya. Journal of AIDS & clinical research. 2012 Jan 1;3(7). 10.4172/2155-6113.1000170 PMC3595060 23495369

[29] Croft TN, Marshall AM, Allen CK, Arnold F, Assaf S, Balian S. Guide to DHS statistics. Volume 645. Rockville: ICF; 2018 Aug.

[30] Zou D, Lloyd JE, Baumbusch JL (2020). Using SPSS to analyze complex survey data: a primer. J Mod Appl Stat Methods.

[31] Johnston R, Jones K, Manley D (2018). Confounding and collinearity in regression analysis: a cautionary tale and an alternative procedure, illustrated by studies of british voting behaviour. Qual Quant.

[32] Mandiwa C, Namondwe B, Munthali M (2021). Prevalence and correlates of comprehensive HIV/AIDS knowledge among adolescent girls and young women aged 15–24 years in Malawi: evidence from the 2015–16 Malawi demographic and health survey. BMC Public Health.

[33] Frimpong JB, Budu E, Adu C et al. Comprehensive HIV/AIDS knowledge and safer sex negotiation among adolescent girls and young women in sub-Saharan Africa.Journal of Biosocial Science2021;1–13. 10.1017/S0021932021000493 34558397

[34] Kawuki J, Sserwanja Q, Mukunya D, Sepenu AS, Musaba MW. Prevalence and factors associated with sexual violence among women aged 15–49 years in rural Uganda: evidence from the Uganda Demographic and Health Survey 2016.Public health. 2021 Jul1;196:35–42. 10.1016/j.puhe.2021.05.004 34139607

[35] Ochako R, Ulwodi D, Njagi P, Kimetu S, Onyango A. Trends and determinants of Comprehensive HIV and AIDS knowledge among urban young women in Kenya. AIDS Res therapy. 2011 Dec;8(1):1–8. 10.1186/1742-6405-8-11 PMC3060857 21375746

[36] Estifanos TM, Hui C, Tesfai AW, Teklu ME, Ghebrehiwet MA, Embaye KS, Andegiorgish AK. Predictors of HIV/AIDS comprehensive knowledge and acceptance attitude towards people living with HIV/AIDS among unmarried young females in Uganda: a cross-sectional study. BMC Womens Health. 2021 Dec;21(1):1–3. 10.1186/s12905-021-01176-w PMC7836492 33499860

[37] Abate BB, Kassie AM, Reta MA, Ice GH, Haile ZT. Residence and young women’s comprehensive HIV knowledge in Ethiopia. BMC Public Health. 2020 Dec;20(1):1–0. 10.1186/s12889-020-09687-1 PMC7585223 33097014

[38] Fenny AP, Crentsil AO, Asuman D (2017). Determinants and distribution of comprehensive HIV/AIDS knowledge in Ghana. Glob J Health Sci.

[39] Wetheridge L, Girls’. and women’s literacy with a lifelong learning perspective: issues, trends and implications for the Sustainable Development Goals. ED-2016/WS/20, https://unesdoc.unesco.org/ark:/48223/pf0000244959. Accessed 12th Aug 2022.

[40] News Global Perspective Human Stories. Literacy has empowering effect on women, UN officials say. 2010. https://news.un.org/en/story/2010/09/350122-literacy-has-empowering-effect-women-un-officials-say. Accessed 12th Aug 2022.

[41] Darteh EK. Individual and contextual predictors of comprehensive HIV and AIDS knowledge among young females in Ghana. African Journal of AIDS Research. 2020 Jul 2;19(3):222 – 30. 10.2989/16085906.2020.1802307 32892711

[42] Bertrand JT, O’Reilly K, Denison J, Anhang R, Sweat M. Systematic review of the effectiveness of mass communication programs to change HIV/AIDS-related behaviors in developing countries. Health education research. 2006 Aug 1;21(4):567 – 97. 10.1093/her/cyl036 16847044

[43] Maksymowicz M, Machowiec PA, Ręka G, Niemirski D, Piecewicz-Szczęsna H. Use of mobile phones by youth regarding the potential health consequences–a survey study. Journal of Education, Health and Sport. 2020 Dec 13;10(12):107 – 17.

[44] Hysing M, Pallesen S, Stormark KM, Jakobsen R, Lundervold AJ, Sivertsen B. Sleep and use of electronic devices in adolescence: results from a large population-based study. BMJ open. 2015 Jan 1;5(1):e006748. 10.1136/bmjopen-2014-006748 PMC4316480 25643702

[45] Xie YJ, Cheung DS, Loke AY, Nogueira BL, Liu KM, Leung AY, Tsang AS, Leong CS, Molassiotis A. Relationships between the usage of televisions, computers, and mobile phones and the quality of sleep in a Chinese population: Community-based cross-sectional study. Journal of medical Internet research. 2020 Jul 7;22(7):e18095. 10.2196/18095 PMC7380995 32369439

[46] Mwaura J, Carter V, Kubheka BZ. Social media health promotion in South Africa: Opportunities and challenges. African Journal of Primary Health Care and Family Medicine. 2020 Jan 1;12(1):1–7. 10.4102/phcfm.v12i1.2389 PMC7433217 32787400

[47] Albalawi Y, Sixsmith J. Identifying Twitter influencer profiles for health promotion in Saudi Arabia. Health promotion international. 2017 Jun 1;32(3):456 – 63. 10.1093/heapro/dav103 26516181

[48] Agegnehu CD, Geremew BM, Sisay MM, Muchie KF, Engida ZT, Gudayu TW, Weldetsadik DS, Liyew AM. Determinants of comprehensive knowledge of HIV/AIDS among reproductive age (15–49 years) women in Ethiopia: further analysis of 2016 ethiopian demographic and health survey. AIDS Res Therapy. 2020 Dec;17(1):1–9. 10.1186/s12981-020-00305-z PMC7425582 32787881

[49] Centers for Disease Control (CDC. Public Health Service guidelines for counseling and antibody testing to prevent HIV infection and AIDS. MMWR. Morbidity and mortality weekly report. 1987 Aug 14;36(31):509 – 15. 3112540

[50] Yehadji D. Urban-rural disparities in HIV related knowledge, behavior and attitude in Burkina Faso: Evidence from Burkina Faso Demographic and Health Survey 2010. 2015. https://www.researchgate.net/publication/281686077_Urban-rural_disparities_in_HIV_related_knowledge_behavior_and_attitude_in_Burkina_Faso_Evidence_from_Burkina_Faso_Demographic_and_Health_Survey_2010. Accessed 12th Aug 2022.

[51] Yaya S, Ghose B, Udenigwe O, Shah V, Hudani A, Ekholuenetale M. Knowledge and attitude of HIV/AIDS among women in Nigeria: a cross-sectional study. European journal of public health. 2019 Feb 1;29(1):111-7. 10.1093/eurpub/cky131 30053009

[52] Veinot TC, Harris R. Talking about, knowing about HIV/AIDS in Canada: a rural-urban comparison. J Rural Health. 2011 Jun;27(3):310–8. 10.1111/j.1748-0361.2010.00353.x 21729159

[53] Hazarika I. Knowledge, attitude, beliefs and practices in HIV/AIDS in India: identifying the gender and rural–urban differences. Asian Pacific Journal of Tropical Medicine. 2010 Oct 1;3(10):821-7.

[54] Idler EL, editor. Religion as a social determinant of public health. Oxford University Press, USA;; 2014. 10.1093/ACPROF:OSO/9780199362202.001.0001.

[55] Mohamed N, Tackling AIDS, Through. Islam? 2005. https://www.islamonline.net/servlet/Satellite?c=Article_C&cid=1157962465531&pagename=Zone-English-HealthScience%2FHSELayout. Accessed 12th Aug 2022.

[56] Catholic News Agency. United Church of Christ committee recommends condom distribution at churches. 2009. https://www.catholicnewsagency.com/news/15505/united-church-of-christ-committee-recommends-condom-distribution-at-churches. Accessed 12 Aug 2022.

[57] The Lutheran World Federation. India’s Lutheran churches fight AIDS stigma | The Lutheran World Federation. 2016. https://www.lutheranworld.org/news/indias-lutheran-churches-fight-aids-stigma. Accessed 12th Aug 2022.

[58] Zou J, Yamanaka Y, John M, Watt M, Ostermann J, Thielman N. Religion and HIV in Tanzania: influence of religious beliefs on HIV stigma, disclosure, and treatment attitudes. BMC Public Health. 2009 Dec;9(1):1–2. 10.1186/1471-2458-9-75 PMC2656538 19261186

[59] Mensah C. Access to Health Insurance and Health-Seeking Behavior in a Nigerian Suburban Community. Walden Dissertations and Doctoral Studies. 2020. https://www.proquest.com/docview/2442570158?pq-origsite=gscholar&fromopenview=true. Accessed 12 Aug 2022.

